# Dysregulated T helper cytokines in serum and peritoneal fluid of women with endometriosis-related infertility

**DOI:** 10.2478/abm-2026-0028

**Published:** 2026-08-31

**Authors:** Fadhil Ahsan, Nanda Yuli Rahmawati, M. Y. Ardianta Widyanugraha, Arif Tunjungseto, Sri Ratna Dwiningsih, Ashon Sa'adi, Budi Santoso

**Affiliations:** Department of Obstetrics and Gynecology, Faculty of Medicine, Universitas Airlangga, Surabaya 60132, Indonesia; Department of Obstetrics and Gynecology, University Hospital of Würzburg, Würzburg 97080, Germany; Doctoral Program of Medical Science, Faculty of Medicine, Universitas Airlangga, Surabaya 60132, Indonesia

**Keywords:** biomarker, endometriosis, gynecologic inflammation, infertility, interleukin, reproductive immunology, T helper cytokine, Th1/Th2 balance, Th2 immune response

## Abstract

**Background:**

Endometriosis is a chronic inflammatory condition and is one of the leading causes of infertility. The role of T helper (Th) cytokines in infertility remains largely unexamined, despite their recognized importance in endometriosis.

**Objectives:**

To investigate the serum and peritoneal fluid levels of IL-4, IL-5, IL-10, IL-17A, IL-17F, and IFN-γ in women with endometriosis and to explore their correlations with disease severity.

**Methods:**

Eighty-seven infertile women undergoing diagnostic laparoscopy were included, comprising 44 endometriosis patients and 43 controls with benign gynecologic disorders. Serum and peritoneal cytokines were measured using Enzyme-linked immunosorbent assay. Statistical analyses assessed differences and correlations with clinical parameters.

**Results:**

Both serum and peritoneal fluid levels of IL-4 and IL-5 were markedly higher in endometriosis patients compared to controls (*P* < 0.001). Although serum IL-10 levels did not show significant differences, there was an increase in peritoneal fluid (*P* < 0.001). Among those with endometriosis, serum levels of IL-4, IL-10, IL-17A, and IL-17F exhibited a positive correlation with the revised American Society for Reproductive Medicine score, while IL-17F indicated an inverse correlation with the Endometriosis Fertility Index (EFI) scores (*r* = −0.422, *P* = 0.004). Receiver operating characteristic curve analysis demonstrated that IL-4 and IL-5 effectively distinguished endometriosis cases from controls, with IL-17F also providing significant predictive value for EFI scores (*P* = 0.015).

**Conclusions:**

Elevated IL-4 and IL-5 levels in endometriosis suggest a Th2-skewed immune response. Meanwhile, the correlation of IL-17A with disease severity and the inverse relationship of IL-17F with fertility scores highlight the complex role of the Th17 response. These findings enhance understanding of cytokine dysregulation in endometriosis and may guide future therapeutic strategies.

Endometriosis is a common gynecological condition that impacts about 10% of women in their reproductive years, marked by endometrial-like tissue found outside the uterus [1]. This ectopic tissue causes chronic pelvic pain, dysmenorrhea, and infertility, severely affecting women's quality of life [1, 2]. Despite extensive research, the precise pathogenesis of endometriosis remains elusive. However, accumulating evidence suggests that dysregulation of the immune system plays a crucial role in its development and progression [1, 3, 4]. Understanding the complex interactions between immune responses and endometriosis is essential for developing effective treatments and improving patient outcomes [1, 3].

Cytokines, small proteins released by immune cells, are crucial in mediating and regulating immune responses. In the context of endometriosis, these cytokines are essential to the inflammatory processes characteristic of the disease [4, 5]. Among these, T helper (Th) type 2 cytokines, such as IL-4 and IL-5, are particularly relevant because they promote inflammation, tissue remodeling, and immune responses, contributing to disease progression [5, 6]. Earlier research indicates that women with endometriosis show higher IL-4 levels than healthy controls, pointing to a Th2-skewed immune response that could worsen inflammation [5,6,7]. Findings on IL-5 have been mixed, with some studies indicating elevated levels in endometriosis [8, 9], while others show no significant differences [7, 10]. IL-10, an anti-inflammatory cytokine, also shows mixed results, with some studies reporting increased levels [7, 11,12,13,14] and others showing no significant differences [9]. IL-10's role is complex, as it can suppress and promote inflammation depending on the context [11, 14]. Further investigation into Th2-related cytokines, particularly in infertile women, is warranted.

Th17 cytokines, particularly IL-17A and IL-17F, play critical roles in autoimmunity and chronic inflammation [15]. IL-17A is elevated in endometriosis and is associated with disease severity [16]. Hirata et al. [17] reported that IL-17A induces IL-8 secretion and cell proliferation of endometriotic stromal cells. In contrast, IL-17F has been underexplored and is often studied within the total IL-17 context [16, 17]. IL-17 modulates macrophage polarization in endometriosis and endometrial stromal cell growth, and increased peritoneal Th17 cells are associated with disease severity [17,18,19,20]. IL-17 in endometriosis may contribute to increased inflammation and potentially affect fertility [21]. IFN-γ, a Th1 cytokine, is crucial for enhancing antigen presentation and promoting Th1 polarization [22]. Its role in endometriosis remains debated, with studies reporting elevated levels contributing to inflammation or reduced/insignificant differences [23].

Given the prevalence of endometriosis and its profound impact on women's health, especially in the context of infertility cases, further research into the roles of Th cytokines is essential for understanding the inflammatory processes driving the disease. Here, we report a detailed evaluation of serum and peritoneal fluid levels of IL-4, IL-5, IL-10, IL-17A, IL-17F, and IFN-γ in infertile women with endometriosis compared to control subjects. We also explore the correlations between these cytokines and clinical parameters.

## Methods

### Study design

The Ethical Review Committee of the Faculty of Medicine, Airlangga University, Surabaya, Indonesia (No. 56/EC/KEPK/FKUA/2022) approved this observational study protocol. A total of 87 patients who underwent diagnostic laparoscopy were enrolled consecutively and signed informed consent. Among them, 44 patients had laparoscopy-confirmed endometriosis and were assigned to the case group, while 43 non-endometriosis patients were assigned to the control group. The control group consisted of non-endometriosis women with a single benign gynecologic disorder related to the fallopian tubes, ovaries, or fibroids. Exclusion criteria included the use of hormonal medications, immunosuppressive drugs, unexplained infertility, and the presence of more than one gynecological disorder. Patients with autoimmune diseases, diabetes mellitus, infections, or malignancies were excluded from the study.

### Endometriosis classification

The stage of endometriosis was determined using the revised American Society for Reproductive Medicine (rASRM) classification [24], which comprises endometriosis and adhesion scores and categorizes the disease into 4 stages (I–IV) based on the extent, location, and severity of the endometriotic lesions. Additionally, the Endometriosis Fertility Index (EFI) scores were calculated to assess fertility prognosis in women with endometriosis [25].

### Collection of peritoneal fluid and serum

Peritoneal fluid was collected from the Douglas pouch area or perivesical space by aspiration needle using laparoscopic direct vision. The sample was placed in a 15 mL sterile centrifuge tube and immediately centrifuged at 3,000 rpm, 4°C for 10 min. The supernatant was then put into 1.5 mL centrifuge tubes. For serum collection, a total of 5 mL blood samples were obtained prior to the laparoscopic examination. The blood samples were collected into BD Vacutainer serum separator tubes (BD, Franklin Lakes, NJ, USA), left at room temperature for 30 min to allow clotting, and further centrifuged at 3,000 rpm, 4°C for 10 min. The serum was isolated in the upper phase and placed into 1.5 mL sterile microtubes. All peritoneal fluid and serum samples were labeled and stored at −80°C until further analysis.

### Enzyme-linked immunosorbent assay

The levels of immune parameters in the serum and peritoneal fluid of patients were measured using Quantikine Enzyme-linked immunosorbent assay (ELISA) kits for human IL-4 (D4050), IL-5 (D5000B), IL-10 (D1000B), IL-17A (D1700), IL-17F (D1700C), and IFN-γ (DIF50) (R&D Systems, Minneapolis, MN, USA), following the manufacturer's instructions. The intra- and inter-assay CVs and sensitivity of the assays are as follows: IL-4 (3.3%, 5.6%, 2 pg/mL), IL-5 (3.8%, 6.1%, 3 pg/mL), IL-10 (4.2%, 7.2%, 1 pg/mL), IL-17A (4.5%, 7.8%, 1 pg/mL), IL-17F (4.9%, 8.3%, 1 pg/mL), and IFN-γ (3.2%, 6.0%, 8 pg/mL). Plates were developed using tetramethylbenzidine substrate (ThermoFisher, Waltham, MA, USA), stopped with 2 N H_2_SO_4_, and read at 450 nm using a microplate reader (BIO-RAD, Hercules, CA, USA). Concentrations of cytokines were measured in picograms per milliliter.

### Statistical analysis

All statistical analyses were conducted using GraphPad Prism version 9 (GraphPad Software, San Diego, CA, USA). A power analysis was performed prior to the study to determine the sample size. Data distribution was assessed using the Shapiro–Wilk normality test. The two-tailed Mann–Whitney *U* test was used to determine the differences between the endometriosis and control groups. The comparison of 3 or more groups was done using the Kruskal–Wallis rank test. Correlation analysis was performed using Spearman's rank test (rho, *r*) between two continuous variables. All results were presented as median and interquartile range. A *P*-value below 0.05 was considered statistically significant. The receiver operating characteristic (ROC) curve analysis was performed to determine the cytokine's sensitivity, specificity, and cut-off value in patients with endometriosis versus the control group.

## Results

### Patient's characteristics

The analysis of baseline characteristics, shown in **Table 1**, revealed no significant differences in age, BMI, or menstrual cycle phase between endometriosis and control groups (*P* > 0.05). Although dysmenorrhea and dyspareunia were more prevalent in the endometriosis group, these differences were not statistically significant (*P* = 0.134 and *P* = 0.229, respectively). The median rASRM endometriosis and adhesion scores for the endometriosis group were 22 and 9.5, respectively. In addition, the median EFI score of endometriosis cases was 5.50.

**Table 1.** Patient's characteristics

**Variables**	**Control (n = 43)**	**Endometriosis (n = 44)**	***P* ^c^**
Age (years)^a^	31 (28–36)	30 (28–33)	0.580
Body mass index (BMI)^a^	22.80 (20.40–25.30)	23.20 (20.40–26.50)	0.544
Dymenorrhea^b,d^			0.134
Yes	16 (37.21%)	24 (54.55%)	
No	27 (62.79%)	20 (45.45%)	
Dyspareunia^b,d^			0.229
Yes	4 (9.30%)	9 (20.46%)	
No	39 (90.69%)	35 (79.54%)	
rASRM score^a^			N/A
Endometriosis score	N/A	22 (8.00–47.50)	
Adhesion score		9.50 (2.25–17.25)	
Total EFI score^a^	N/A	5.50 (4–7)	N/A
Menstrual cycle^b^			0.391
Follicular	17 (39.53%)	22 (50.00%)	
Luteal	26 (60.46%)	22 (50.00%)	

Data are shown as

a median (25%–75% percentile) and

b frequency (percentage).

c Statistical analysis was performed using a two-tailed Mann-Whitney U test.

d Symptoms in the previous month based on anamnesis.

rASRM, revised American Society for Reproductive Medicine; EFI, Endometriosis Fertility Index; N/A: Not applicable.

### Serum and peritoneal fluid cytokine levels

The analysis of serum cytokine levels revealed significant differences between women with endometriosis and control subjects. As shown in **Figure 1A**, IL-4 levels were significantly elevated in the endometriosis group compared to controls (*P* = 0.009). Moreover, IL-5 levels were markedly increased in the endometriosis group compared to controls (*P* < 0.001). In contrast, no significant differences in serum IL-10, IL-17A, IL-17F, and IFN-γ were observed between endometriosis and control groups. In stratifying endometriosis cases by stage (**Supplementary Table 1**), the serum levels of IL-5, IL-10, IL-17F, and IFN-γ showed significant variation across stages, with *P*-values of 0.026, 0.019, 0.017, and 0.007, respectively. Serum levels of IL-5 and IFN-γ exhibited significant differences between stage IV and stage III. Specifically, IL-5 levels were elevated in stage IV (*P* = 0.037), while IFN-γ levels were higher in stage III (*P* = 0.022). In comparing stage I endometriosis with higher stages, serum IFN-γ levels were significantly higher in stage III than in stage I (*P* = 0.025). Similarly, serum IL-17A was elevated in stage III relative to stage I (*P* = 0.038), and serum IL-17F was also higher in stage IV compared to stage I (*P* = 0.049). Serum IL-10 at stage III was higher compared to stage II cases (*P* = 0.031). Peritoneal fluid cytokines generally did not show significant differences across stages, except for IFN-γ, where significant differences were observed in serum and peritoneal fluid. Stage IV had significantly higher peritoneal IFN-γ compared to stage I cases (*P* = 0.023).

**Figure 1.**
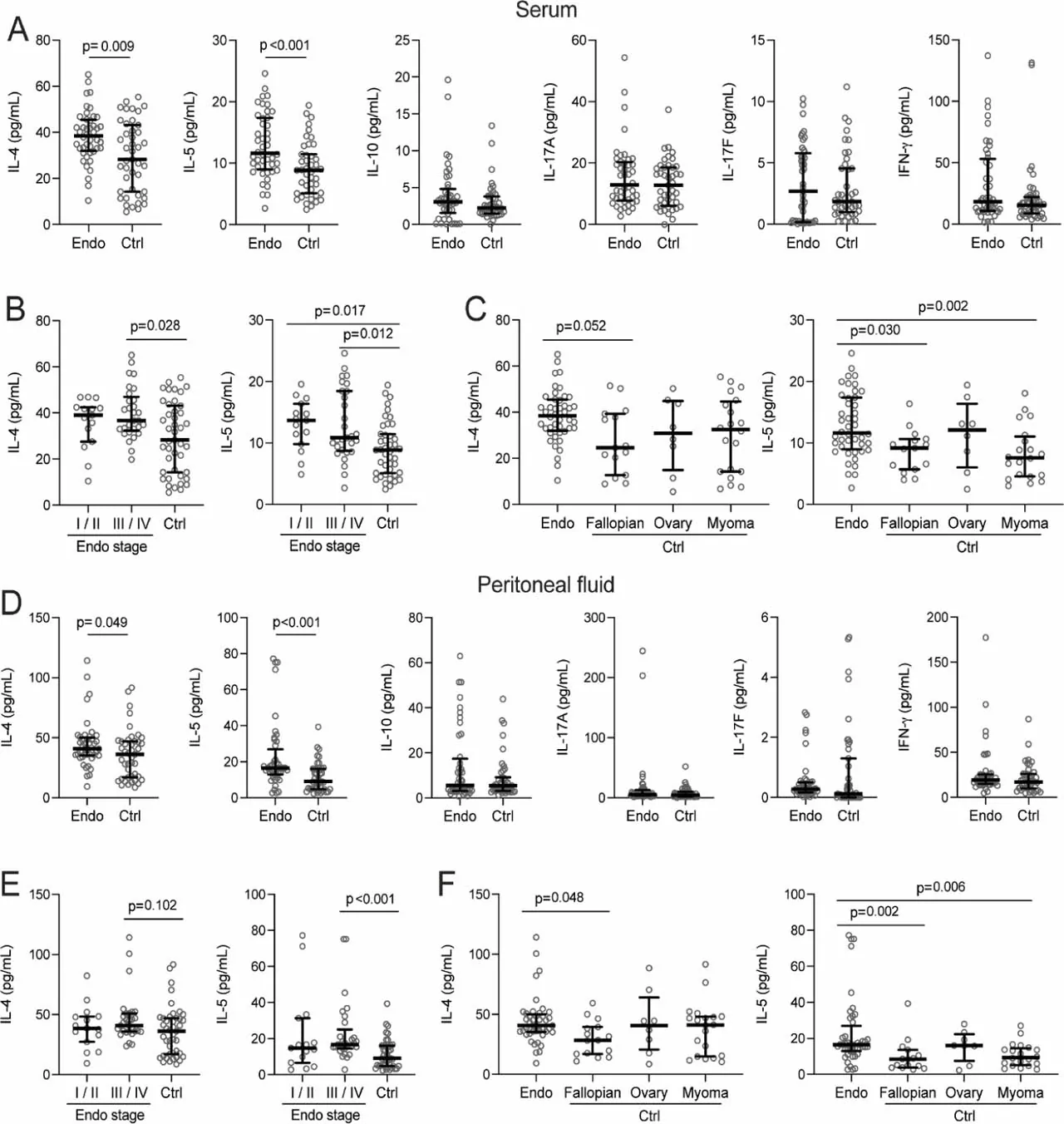
Serum and peritoneal Th cytokine levels in endometriosis and gynecologic control patients. Scatter plots of (**A**) serum and (**D**) peritoneal IL-4, IL-5, IL-10, IL-17A, IL-17F, and IFN-γ levels in endometriosis and control groups. (**B**) Serum and (**E**) peritoneal IL-4 and IL-5 levels stratified by disease stage (stage I/II and III/IV) compared to control cases. (**C**) Serum and (**F**) peritoneal IL-4 and IL-5 levels in different control subgroups-fallopian, ovary, and myoma-compared to endometriosis. A dot represents each subject. *P*-values are indicated. Endo, Endometriosis group. Ctrl, Control group. Th, T helper.

Subsequently, we examined differences in serum Th cytokines between the stages of endometriosis and the control group (**Figure 1B**). The increase in IL-4 was consistently observed in the later stages of endometriosis (stages III/IV) compared to the controls (*P* = 0.028). Additionally, elevated levels of IL-5 were noted in both the early and late stages of the disease versus the controls (*P* = 0.017 and *P* = 0.012, respectively). Among different types of gynecologic controls, the lowest median IL-4 levels were found in fallopian-related infertility cases (24.56 pg/mL) (**Figure 1C**). Control subgroups, especially fallopian and myoma-related cases, exhibited significantly lower IL-5 levels than endometriosis cases (9.16 pg/mL, *P* = 0.030 and 7.58 pg/mL, *P* = 0.002, respectively) (**Figure 1C**).

Extending the findings from serum analysis, we further analyzed cytokine levels in peritoneal fluid. As shown in **Figure 1D**, IL-4 levels were significantly elevated in the endometriosis group compared to controls (*P* = 0.049). Similarly, IL-5 levels were markedly increased in the endometriosis group (*P* < 0.001). However, there were no significant differences for other cytokines between endometriosis and control groups in peritoneal fluid. When categorizing endometriosis cases by stages (**Figure 1E**), the median IL-4 levels at stage III/IV endometriosis were higher but did not differ significantly from the control (40.79 versus 36.09 pg/mL, *P* = 0.102). In contrast, IL-5 levels were significantly elevated in late stages (III/IV) endometriosis compared to controls (16.59 versus 9.01 pg/mL, *P* < 0.001). **Figure 1F** compares different types of gynecologic controls versus endometriosis cases. The lowest median IL-4 levels were found in fallopian-related infertility cases (28.50 pg/mL, *P* = 0.048). IL-5 levels were significantly lower in fallopian (8.43 pg/mL, *P* = 0.002) and myoma-related pathology (9.36 pg/mL, *P* = 0.006) than in endometriosis cases (16.43 pg/mL).

### Correlation between Th cytokines and rASRM scores

To assess the relationship between cytokine levels and endometriosis severity, we examined correlations with rASRM scores (**Figure 2**). IL-4 levels were positively correlated with rASRM endometriosis scores (*r* = 0.296, *P* = 0.051) and adhesion scores (*r* = 0.350, *P* = 0.020) (**Figure 2A**). IL-5 levels, however, showed no significant correlations with both rASRM endometriosis scores (*r* = 0.209, *P* = 0.173) and adhesion scores (*r* = 0.245, *P* = 0.108) (**Figure 2B**). In **Figure 2C**, IL-10 levels exhibited a significant negative correlation with rASRM endometriosis scores (*r* = −0.414, *P* = 0.005) and adhesion scores (*r* = −0.336, *P* = 0.026) (**Figure 2C**).

**Figure 2.**
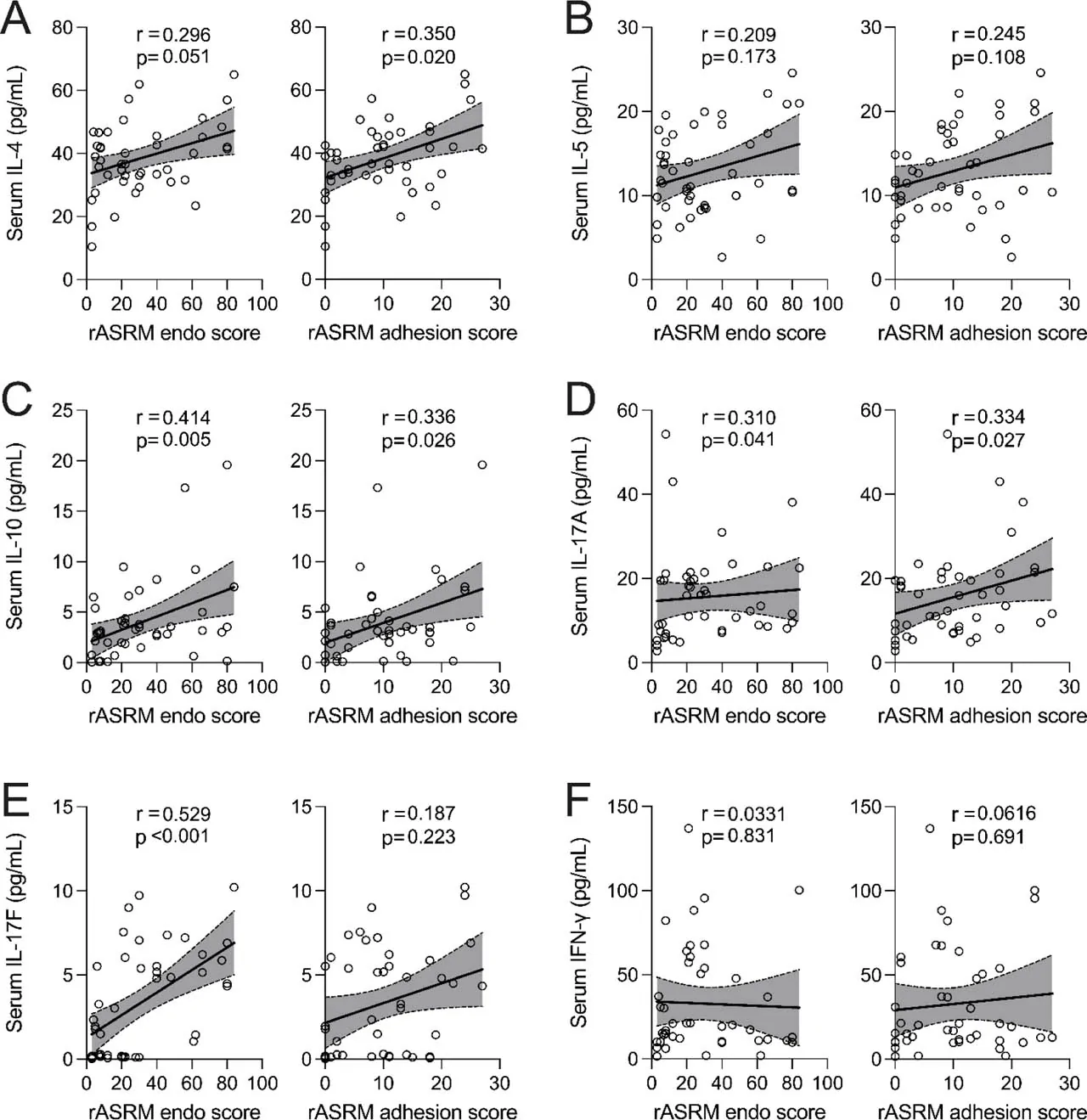
Spearman correlation analysis between serum cytokine levels and rASRM scores. Correlations between **(A)** IL-4, **(B)** IL-5, **(C)** IL-10, **(D)** IL-17A, **(E)** IL-17F, and **(F)** IFN-γ levels with rASRM endometriosis and adhesion scores. Rho (*r*) and *P*-values are indicated. rASRM, revised American Society for Reproductive Medicine.

For Th17- and Th1-related cytokines, IL-17A levels had positive correlations with rASRM endometriosis scores (*r* = 0.310, *P* = 0.041) and adhesion scores (*r* = 0.334, *P* = 0.027) (**Figure 2D**). IL-17F showed a strong positive correlation with rASRM endometriosis scores (*r* = 0.529, *P* < 0.001) but no significant correlation with adhesion scores (*r* = 0.187, *P* = 0.223) (**Figure 2E**). IFN-γ levels did not exhibit significant correlations with either rASRM endometriosis scores (*r* = 0.0331, *P* = 0.831) or adhesion scores (*r* = 0.0616, *P* = 0.691) (**Figure 2F**).

### Correlation between Th cytokines and menstrual cycle

To evaluate the potential influence of menstrual cycle phase, we compared Th cytokine levels between follicular and luteal phases across all participants, as well as within the endometriosis and control groups (**Supplementary Table 2**). No significant differences were observed for any Th cytokines except for serum IL-17A, which was significantly elevated during the follicular phase compared to the luteal phase in the overall sample (*P* = 0.036). This difference was consistent across groups and did not significantly interact with disease status. Furthermore, the luteal phase showed a trend of higher IL-4, IL5, and IL-10, as well as lower IL-17A, IL-17F, and IFN-γ compared to the follicular phase in serum and peritoneal fluid. However, most of them were not statistically significant except serum IL-17A.

### Correlation between Th cytokines and EFI scores and their diagnostic performance

To evaluate the potential impact of cytokine levels on fertility outcomes, we analyzed their correlation with EFI scores. As shown in **Figure 3**, IL-4 and IL-5 levels showed no significant correlation with EFI scores (IL-4: *P* = 0.124; IL-5: *P* = 0.271). IL-10 and IL-17A levels tended to correlate negatively with EFI scores, but these correlations were insignificant (IL-10: *P* = 0.053; IL-17A: *P* = 0.089). In contrast, IL-17F levels exhibited a significant negative correlation with EFI scores (*r* = −0.422, *P* = 0.004). IFN-γ levels showed no significant correlation with EFI scores (*r* = −0.152, *P* = 0.326). When endometriosis cases were categorized into two EFI score groups based on the median value, those with an EFI score exceeding 5 exhibited markedly lower concentrations of IL-17F in serum and peritoneal fluid (*P* = 0.030 and *P* = 0.015, respectively) (**Supplementary Table 3**). Conversely, other cytokine concentrations showed no significant differences in relation to the EFI scores.

**Figure 3.**
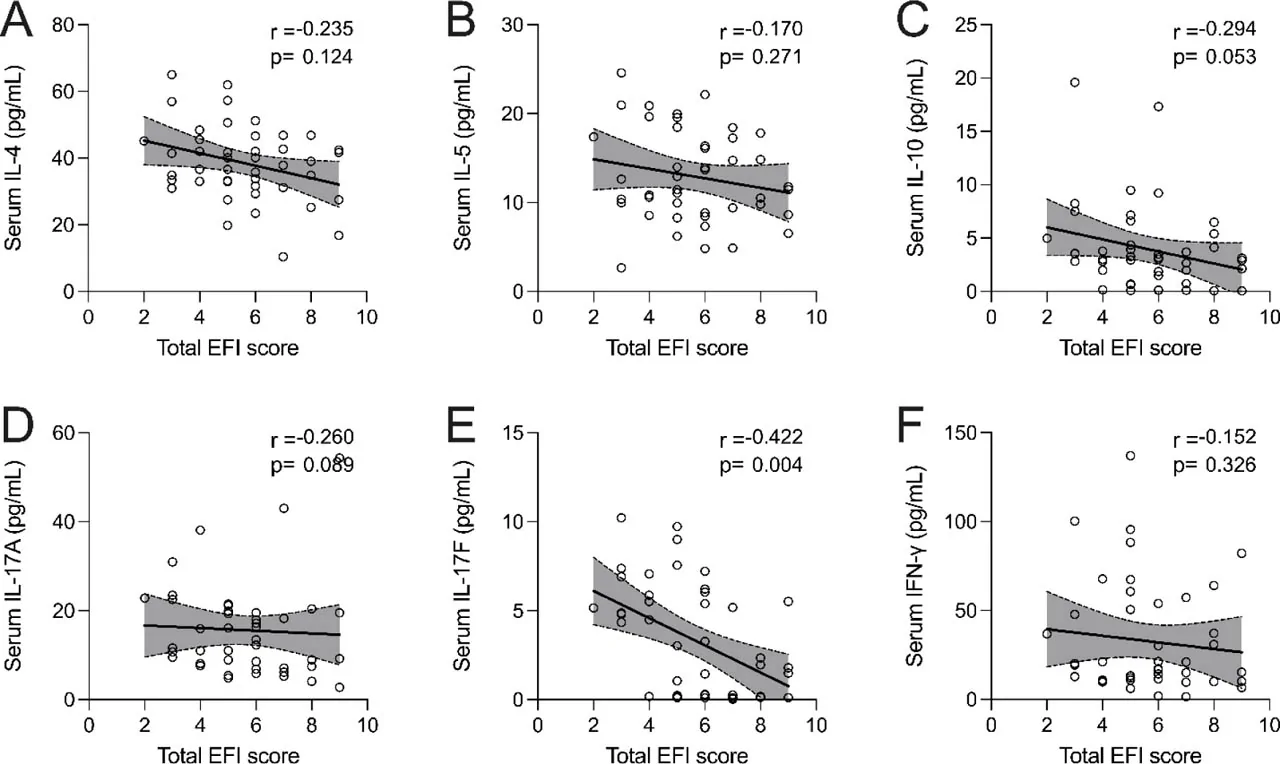
Spearman correlation analysis between serum cytokine levels and EFI scores. Correlations between **(A)** IL-4, **(B)** IL-5, **(C)** IL-10, **(D)** IL-17A, **(E)** IL-17F, and **(F)** IFN-γ levels with the total EFI scores. Rho (*r*) and *P*-values are indicated. EFI, Endometriosis Fertility Index.

To determine the diagnostic value of IL-4 and IL-5 for endometriosis and the predictive value of IL-10 and IL-17F for EFI scores, we performed ROC curve analysis (**Figure 4**). IL-4 had an area under the curve (AUC) of 0.662 (*P* = 0.009), and IL-5 had an AUC of 0.714 (*P* < 0.001) in distinguishing endometriosis from controls (**Figure 4A**).

**Figure 4.**
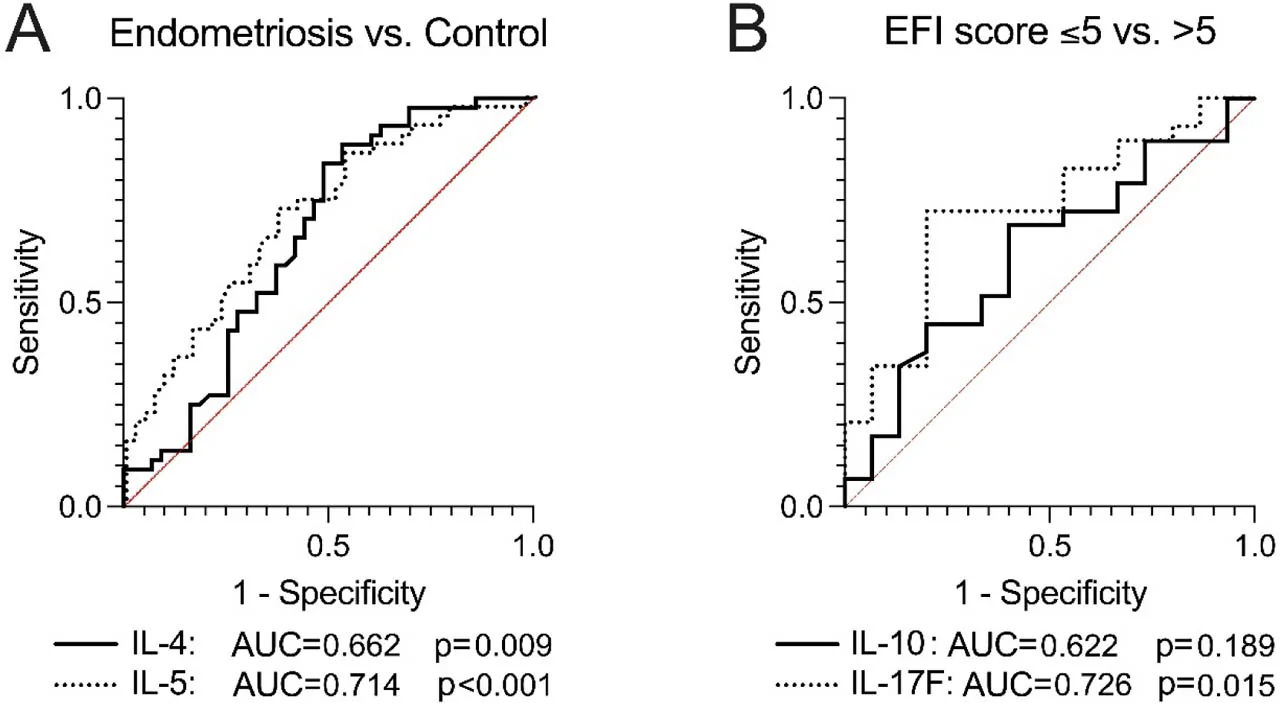
ROC curve analysis for cytokines in serum. **(A)** Serum IL-4 and IL-5 in differentiating endometriosis from control cases. **(B)** Serum IL-10 and IL-17F in differentiating EFI scores above 5 versus 5 or below. The AUC and *P*-values are indicated. AUC, area under the curve; EFI, Endometriosis Fertility Index; ROC, receiver operating characteristic.

For predicting EFI scores, IL-10 had an AUC of 0.622 (*P* = 0.189), indicating limited diagnostic value (**Figure 4B**). In contrast, IL-17F showed a significant predictive value with an AUC of 0.726 (*P* = 0.015) for EFI scores (**Figure 4B**), indicating a potential predictor of fertility outcomes in affected women.

## Discussion

This study provides significant insights into the role of Th cytokines in the immunopathology of endometriosis-related infertility, highlighting substantial differences and correlations with clinical parameters. Key findings include elevation of serum and peritoneal IL-4 and IL-5 in endometriosis, particularly in severe cases, compared to control subjects, and positive correlation of IL-17A and IL-17F with disease severity among endometriosis cases. Our study presents 3 novel insights into cytokine expression among infertile women, both with and without endometriosis, and their fertility outcomes. It links cytokine profiles to fertility potential using the EFI and highlights IL-17F as a potential biomarker for fertility impairment, advancing the clinical assessment of endometriosis.

Our study found significantly higher levels of IL-4 and IL-5 in endometriosis patients' serum and peritoneal fluid, particularly in severe cases. This finding aligns with the hypothesis that endometriosis is associated with a Th2-dominant immune response [4, 5, 8]. Elevated IL-4 and IL-5 levels were also observed in early-stage endometriosis compared to controls, suggesting that these cytokines may be involved earlier in the disease development. The lowest median levels of IL-4 and IL-5 were found in control cases related to fallopian tube disorders, indicating that these cytokines are particularly elevated in endometriosis. The persistence of elevated IL-4 and IL-5 levels across early and late endometriosis stages suggests that the Th2-like immune response is an early and continuous feature of endometriosis progression. The positive correlation between serum IL-4 and rASRM adhesion scores further supports the role of IL-4 in the formation of adhesions. The development of endometriosis involves the proliferation, growth, adhesion, and invasion of endometriotic tissue, processes driven by locally produced IL-4 [4, 11]. The ROC curve analysis showed that serum IL-4 and IL-5 could discriminate between endometriosis and control cases, although the AUC values are still below 0.8, a common requirement of a good biomarker [26].

Despite no significant differences in serum IL-17A and IL-17F levels between endometriosis patients and controls, these cytokines were positively correlated with rASRM endometriosis scores among endometriosis cases. Previous studies have reported similar findings, indicating that IL-17A promotes inflammation and the progression of endometriosis [16,17,18]. Th17 cytokines contribute to neutrophil recruitment, inflammatory amplification, and lesion persistence [16,17,18]. Interestingly, only IL-17A was correlated with rASRM adhesion scores, underscoring its role in developing pelvic adhesions [16, 19]. Furthermore, IL-17F was inversely correlated with the EFI score, indicating that higher levels of IL-17F are associated with poorer fertility outcomes. A previous report by Roomandeh et al. [27] revealed that women with unexplained recurrent spontaneous abortion had higher serum IL-17 compared to healthy controls, indicating the role of IL-17 in fertility outcomes. IL-17F promotes tissue damage and local inflammation [15, 16], which may play a critical role in endometrial receptivity, leading to reduced fertility in endometriosis. The ROC curve analysis demonstrated that serum IL-17F could differentiate EFI scores above 5 versus 5 or below, highlighting its potential as a fertility biomarker in endometriosis patients.

Serum IL-10 levels did not differ significantly between endometriosis patients and controls. However, IL-10 was positively correlated with both rASRM endometriosis and adhesion scores. This suggests that while systemic IL-10 levels might not be indicative of endometriosis presence, local IL-10 production may still play a role in modulating inflammation and tissue remodeling within the peritoneal environment [11,12,13,14]. Previous studies have reported varying findings regarding IL-10 levels in endometriosis, reflecting its complex role in immune regulation and context-dependent effects [7, 11].

No significant differences between endometriosis patients and controls were found in serum and peritoneal IFN-γ levels. Additionally, IFN-γ did not correlate with rASRM scores, suggesting that this cytokine may not play a central role in the pathogenesis of endometriosis-related infertility or its severity. Previous findings on IFN-γ levels have revealed contradictory results in endometriosis [23]. The lack of significant differences in our study may be due to differences in the study population, disease stages, or methodological variations. The variability in IFN-γ findings suggests that while it may play a role in the immune response to endometriosis-related infertility, its impact may not be as pronounced or consistent as other cytokines like IL-4 and IL-17A.

The significance of cytokines in endometriosis-related infertility is highlighted, underscoring the necessity for further investigation to comprehensively understand these mechanisms. The significance of cytokine levels in the follicular fluid may impact the in vitro fertilization-embryo transfer outcomes, particularly relevant in the context of endometriosis-associated infertility [28]. Additionally, inflammatory factors can influence the success of embryo implantation by disrupting the balance between Th1/Th2 and Th17/regulatory T cells and causing imbalances in uterine natural killer cells and macrophages [29]. Th cytokines can directly and indirectly affect endometriosis and infertility. IL-4 promotes differentiation of naïve CD4+ T cells into Th2 cells, fostering an inflammatory environment that helps tissue remodeling and fibrosis while inhibiting cytotoxic T cell activation, allowing ectopic endometrial tissue to persist [4, 6]. Elevated IL-5 levels worsen localized inflammation in the peritoneal cavity, causing tissue damage and interfering with embryo implantation, negatively impacting fertility [9]. Indirectly, IL-10, primarily anti-inflammatory, can lead to insufficient immune responses to ectopic lesions, leading to chronic inflammation that reduces endometrial receptivity [9, 11]. IL-17A and IFN-γ contribute to inflammation and oxidative stress, harming ovarian function and oocyte quality and disrupting hormonal signaling, creating an unfavorable environment for implantation [16, 22].

The distinct cytokine profiles observed in this study highlight the multifaceted nature of immune dysregulation in endometriosis [3, 4, 28]. The Th2 immune response indicated by elevated IL-4 and IL-5 levels suggests that endometriosis may involve a shift toward a humoral immune response, potentially facilitating the persistence and growth of ectopic endometrial tissues [3,4,5,6, 28]. The role of IL-17A and IL-17F in disease severity and fertility underscores the importance of the Th17 axis in endometriosis pathology [16]. Our study found that IL-17F exhibited compartmentalized behavior, with distinct levels in peritoneal fluid compared to serum, reflecting its specific cellular sources and regulatory mechanisms. This indicates a localized role in the inflammatory response associated with endometriosis, primarily produced by immune cells such as Th17 cells and ILC3s, as well as activated macrophages [15]. The regulation of IL-17F expression is influenced by inflammatory cytokines and the presence of ectopic endometrial tissue, which can enhance its production in the peritoneal cavity [16]. This compartmentalization highlights the need to consider cytokine sources and regulatory context in assessing their roles in endometriosis. Further research should clarify the mechanisms behind these cytokine changes and their impact on disease progression and treatment outcomes.

Given the known effects of hormonal fluctuations on immune responses throughout the menstrual cycle, which may affect the inflammatory profile [30], we assessed Th cytokine levels by cycle phase in this study. Our analysis revealed that serum IL-17A was significantly higher in the follicular phase across all subjects, aligning with prior studies reporting elevated IL-17A during this phase [31, 32]. This may reflect estrogen-driven Th17 activation [32], contributing to a more pro-inflammatory state in the follicular phase and potentially influencing infertility. In contrast, no significant menstrual phase-related differences were observed for other Th cytokines, including IL-4, IL-5, IL-13, IL-10, or IFN-γ, consistent with previous findings suggesting minimal or no variation in these cytokines across the cycle [30, 33,34,35]. Furthermore, the distribution of follicular and luteal phases did not differ significantly between groups, and group comparisons remained unaffected by menstrual phase, suggesting limited confounding from the menstrual cycle in our study.

Several limitations should also be considered when interpreting the findings of this study. The sample size was relatively small, potentially limiting generalizability, and future studies with larger cohorts are needed for confirmation. The cross-sectional design precludes conclusions about causality, requiring longitudinal studies to determine if cytokine changes precede or result from endometriosis. While elevated IL-4 and IL-5 levels in endometriosis patients point to a connection with Th2-associated pathology and disease severity, additional studies are required to confirm causality. Potential confounding factors such as hormonal treatments, diet, and lifestyle were not accounted for, which could influence cytokine levels. Finally, the study focused on peripheral blood and peritoneal fluid samples without examining local tissue cytokine production, which might provide more direct insights into the inflammatory processes within endometriotic lesions.

## Conclusion

Elevated levels of IL-4 and IL-5 suggest a Th2-skewed immune response in endometriosis, while correlations of IL-17A and IL-17F with disease severity and fertility underscore the complex role of Th17 cells. The findings emphasize the significance of cytokine dysregulation in the pathogenesis of endometriosis and its implications for clinical outcomes. These results facilitate future research endeavors focused on developing targeted therapeutic strategies to modulate cytokine profiles, which may enhance the management of endometriosis-associated infertility and other related symptoms.

## Figures and Tables

**Table S1.** Th cytokine levels in endometriosis stages

**Cytokine (Source)^a^**	**Endometriosis**				***P* ^b^**
	**Stage I (n = 6)**	**Stage II (n = 9)**	**Stage III (n = 14)**	**Stage IV (n = 15)**	
IL-4 (Serum)	26.38 (15.22–40.98)	41.68 (36.81–44.54)	33.63 (30.69–42.74)	42.07 (34.90–48.41)	0.059
IL-4 (Peritoneal fluid)	37.81 (15.94–59.50)	38.51 (34.35–46.61)	45.72 (37.77–51.29)	36.36 (33.89–46.86)	0.449
IL-5 (Serum)	10.80 (6.13–15.58)	13.91 (12.19–16.82)	9.13 (8.06–11.79)	16.12 (10.61–20.90)	0.026*
IL-5 (Peritoneal fluid)	8.18 (4.13–42.73)	16.37 (13.42–25.50)	17.96 (15.19–29.79)	16.29 (13.11–20.36)	0.463
IL-10 (Serum)	1.43 (1.07–5.67)	1.98 (1.11–2.98)	3.85 (2.92–6.75)	3.18 (2.65–7.51)	0.019*
IL-10 (Peritoneal fluid)	3.33 (1.19–12.35)	3.79 (3.09–10.62)	3.53 (3.01–8.52)	28.32 (4.77–40.13)	0.064
IL-17A (Serum)	6.38 (3.79–11.60)	9.23 (6.03–32.09)	18.09 (14.85–20.68)	11.64 (8.58–22.54)	0.057
IL-17A (Peritoneal fluid)	2.99 (1.20–7.03)	6.20 (4.88–14.99)	4.48 (3.36–12.79)	6.03 (4.52–14.42)	0.134
IL-17F (Serum)	2.98 (2.10–4.05)	2.27 (2.19–4.38)	4.91 (3.12–7.16)	6.18 (4.35–6.91)	0.017*
IL-17F (Peritoneal fluid)	2.92 (2.62–4.79)	2.44 (2.17–4.53)	2.29 (2.17–4.59)	2.25 (2.15–4.43)	0.311
IFN-γ (Serum)	10.16 (8.42–32.51)	15.31 (13.78–25.65)	58.98 (20.84–73.00)	12.84 (10.27–21.21)	0.007*
IFN-γ (Peritoneal fluid)	11.96 (8.10–17.27)	20.34 (19.30–24.58)	16.03 (13.89–25.91)	21.71 (17.60–47.67)	0.011*

Data is shown as

a median (25%–75% percentile).

b Statistical analysis performed with the Kruskal-Wallis test to rank all data across all groups.

* Significant *P*-value. IL, Interleukin; IFN, Interferon.

**Table S2.** Th cytokine levels and EFI score

**Cytokine (Source)**	**EFI Score ≤5^a^**	**EFI Score >5^a^**	***P* ^b^**
IL-4 (Serum)	33.53 (25.24–43.08)	37.33 (25.29–46.51)	0.314
IL-4 (Peritoneal fluid)	39.57 (29.12–47.92)	37.19 (24.09–50.27)	0.743
IL-5 (Serum)	10.40 (6.87–13.99)	10.18 (6.85–16.13)	0.969
IL-5 (Peritoneal fluid)	13.91 (6.32–18.44)	15.06 (8.85–18.81)	0.816
IL-10 (Serum)	2.65 (1.48–3.97)	2.82 (1.50–4.23)	0.813
IL-10 (Peritoneal fluid)	6.84 (3.05–15.23)	3.89 (2.93–6.56)	0.111
IL-17A (Serum)	11.03 (7.15–18.55)	16.32 (7.95–21.49)	0.120
IL-17A (Peritoneal fluid)	4.13 (2.60–7.18)	5.46 (4.04–10.79)	0.353
IL-17F (Serum)	5.18 (3.59–6.18)	2.68 (2.86–3.49)	0.030*
IL-17F (Peritoneal fluid)	5.48 (3.12–6.87)	2.19 (2.03–2.40)	0.015*
IFN-γ (Serum)	15.25 (10.35–24.66)	18.10 (8.96–65.72)	0.362
IFN-γ (Peritoneal fluid)	18.74 (13.23–25.54)	19.66 (14.12–29.64)	0.565

Data is shown as

a median (25%–75% percentile).

b Statistical analysis was performed using a two-tailed Mann-Whitney U test.

* Significant p-value.

EFI, Endometriosis Fertility Index; IL, Interleukin; IFN, Interferon.

**Table S3.** Th cytokines and menstrual cycle

**Variable^a^**	**Population**	**Follicular phase**	**Luteal phase**	***P* ^b^**
Serum IL-4	All subject Endometriosis Control	39.59 (29.32 – 45.19) 38.38 (30.57 – 45.55) 39.30 (23.93 – 47.18)	33.63 (20.68 – 41.90) 38.44 (33.37 – 45.89) 25.00 (11.45 – 37.04)	0.160 0.638 0.085
Peritoneal IL-4	All subject Endometriosis Control	40.79 (28.24 – 50.98) 37.00 (26.94 – 51.87) 44.45 (28.37 – 55.09)	38.42 (19.53 – 47.86) 43.04 (36.31 – 49.00) 28.58 (14.71 – 46.50)	0.380 0.270 0.080
Serum IL-5	All subject Endometriosis Control	10.61 (6.64 – 14.41) 11.26 (8.79 – 17.50) 8.85 (6.05 – 13.65)	10.33 (6.99 – 14.54) 13.42 (9.58 – 17.56) 8.77 (4.84 – 10.80)	0.609 0.629 0.543
Peritoneal IL-5	All subject Endometriosis Control	15.41 (9.52 – 19.41) 16.02 (9.94 – 23.09) 13.60 (5.63 – 17.95)	13.80 (6.38 – 18.11) 16.84 (14.40 – 29.79) 8.46 (4.73 – 14.12)	0.553 0.392 0.210
Serum IL-10	All subject Endometriosis Control	3.13 (1.28 – 5.21) 3.51 (2.31 – 5.36) 2.21 (1.28 – 5.40)	2.46 (1.57 – 3.72) 2.74 (1.14 – 4.46) 2.33 (1.60 – 3.50)	0.140 0.275 0.690
Peritoneal IL-10	All subject Endometriosis Control	6.25 (2.89 – 12.77) 6.46 (2.88 – 12.87) 5.96 (3.08 – 14.89)	5.13 (3.08 – 12.74) 4.29 (3.14 – 29.60) 5.40 (2.99 – 9.66)	0.877 0.812 0.649
Serum IL-17A	All subject Endometriosis Control	16.32 (9.23 – 21.46) 15.34 (9.17 – 21.75) 16.32 (9.15 – 20.18)	10.43 (6.14 – 17.61) 11.34 (7.08 – 19.39) 9.71 (5.70 – 16.51)	0.036* 0.185 0.100
Peritoneal IL-17A	All subject Endometriosis Control	5.83 (3.03 – 14.42) 5.60 (3.23 – 15.80) 5.83 (2.41 – 13.02)	4.72 (2.64 – 9.13) 5.43 (3.36 – 8.13) 4.01 (1.66 – 9.82)	0.451 0.714 0.663
Serum IL-17F	All subject Endometriosis Control	2.42 (1.20 – 5.87) 3.15 (1.17 – 6.95) 2.39 (1.80 – 5.37)	1.72 (1.12 – 5.30) 1.88 (1.17 – 5.61) 1.57 (1.05 – 3.42)	0.279 0.581 0.434
Peritoneal IL-17F	All subject Endometriosis Control	1.27 (1.12 – 1.52) 1.27 (1.17 – 1.44) 1.28 (1.03 – 1.82)	1.23 (1.01 – 1.57) 1.30 (1.16 – 1.56) 1.04 (1.01 – 1.11)	0.469 0.379 0.221
Serum IFN-γ	All subject Endometriosis Control	18.70 (10.35 – 44.34) 25.22 (10.75 – 59.83) 17.29 (10.35 – 24.80)	14.77 (9.78 – 21.71) 15.96 (10.75 – 35.86) 14.33 (7.88 – 19.21)	0.164 0.542 0.251
Peritoneal IFN-γ	All subject Endometriosis Control	17.60 (14.35 – 26.03) 18.40 (14.72 – 31.08) 17.13 (10.58 – 26.11)	19.03 (12.43 – 25.22) 20.16 (15.23 – 24.41) 17.05 (9.34 – 26.20)	0.633 0.940 0.740

Data is shown as

a median (25%–75% percentile).

b Statistical analysis was performed using a two-tailed Mann-Whitney U test.

* Significant *P*-value. IL, Interleukin; IFN, Interferon.
